# Western Australian food security project

**DOI:** 10.1186/1471-2458-7-214

**Published:** 2007-08-23

**Authors:** Alexandra McManus, Graham Brown, Bruce Maycock

**Affiliations:** 1 Western Australian Centre for Health Promotion Research, Curtin University of Technology, Perth, Australia

## Abstract

**Background:**

The aim of the Western Australian (WA) Food Security Project was to conduct a preliminary investigation into issues relating to food security in one region within the Perth metropolitan area in Western Australia. The first phase of the project involved a food audit in one lower income area that was typical of the region, to identify the range, variety and availability of foods in the region.

**Methods:**

A comprehensive food audit survey was provided to all food outlet owners/operators in one lower socio-economic region within the City of Mandurah (n = 132 outlets). The purpose of the survey was to investigate the range, variety and availability of foods in the Mandurah region as well as examining specific in-store characteristics such as the types of clientele and in-store promotions offered. Surveys were competed for 99 outlets (response rate = 75%).

**Results:**

The range of foods available were predominantly pre-prepared with more than half of the outlets pre-preparing the majority of their food. Sandwiches and rolls were the most popular items sold in the outlets surveyed (n = 51 outlets) followed by pastries such as pies, sausage rolls and pasties (n = 33 outlets). Outlets considered their healthiest food options were sandwiches or rolls (n = 51 outlets), salads (n- = 50 outlets), fruit and vegetables (n = 40 outlets), seafood (n = 27 outlets), meats such as chicken (n = 26 outlets and hot foods such as curries, soups or quiches (n = 23 outlets). The majority of outlets surveyed considered pre-prepared food including sandwiches, rolls and salads, as healthy food options regardless of the content of the filling or dressings used. Few outlets (n = 28%) offered a choice of bread type other than white or wholemeal. High fat pastries and dressings were popular client choices (n = 77%) as were carbonated drinks (n = 88%) and flavoured milks (n = 46%).

**Conclusion:**

These findings clearly indicate the need for further investigation of the impact of access to quality, healthy foods at reasonable cost (food security) on public health, particularly in lower socio-economic areas.

## Background

Food is a fundamental physiological need of life [1]. Lack of food security leading to poor dietary intake, affects psychological well being and increases the risk of chronic illness including cardiovascular disease, type 2 diabetes, overweight and obesity, and some cancers [2-6]. Food security refers to physical and economic access by all people at all times to sufficient, safe, nutritious food to meet their needs and preferences for an active and healthy lifestyle [7]. Four key factors that impact on access to adequate food supply are: economic access; physical access; availability of safe food that is socially and culturally appropriate; and sustained and secure access [4].

Economic access relates to sufficient money or resources to purchase food [4]. The 2001 New South Wales Child Health Survey identified 6.2% of respondents had run out of food and couldn't afford to purchase more on at least one occasion in the previous 12 months. This situation was three times more likely among parents from low income areas compared to other areas [3,5]. Empirical evidence supports this finding and demonstrates that geographical areas of lower socio-economic status are most at risk of food insecurity [3-5,8].

Food insecurity refers to the experience of hunger due to lack of economic or physical access to sufficient healthy food sources [5]. Access to adequate food supply is important in order to consume a healthy diet. Issues of limited access are relevant for groups such as: people on low incomes living in low income areas; the homeless; the physically or mentally disabled; those living in rural or remote areas; and those of culturally and linguistic diverse backgrounds [8]. For many people, physical access such as transport options to alternative shops may be limited, particularly to supermarkets where a wider range of foods of higher nutritional value are available at lower cost than local food outlets [5,9]. Therefore, the local store becomes the primary source of food supply. The capacity for local stores to stock a wide range of foods that are safe, healthy and at competitive prices, is a major challenge. Most small food retailers experience barriers such as insufficient volume of stock orders to receive the advantage of wholesale prices, small margins, high overheads and slow turnover. The slow turnover of goods compromises the quality and safety of foods stocked, hence small local shops relying more heavily on non-perishable foods and high energy dense foods [9,10].

The availability of adequate food sources should be sustained and secure as a basic utility. Anxiety over cost and availability of food and social exclusion may be experienced by people in low income areas [4,11]. Psychological and physical health is at risk for those who consume a diet of low nutritional value. Low socio-economic areas are disadvantaged by availability of healthy food choices and financial security. People in these areas consume more foods high in fat and saturated fat and eat considerably less fruit and vegetables [4]. Studies have linked higher rates of obesity and diet related illness and disease with poverty and geographical areas of disadvantage [8,12-15]. As rates of overweight and obesity rise within the Australian population, the paradox of those at highest risk of food insecurity experiencing the highest rates of overweight and obesity, continues to increase [5,6,12-15].

This study investigated issues relating to food security in one lower socio-economic area in the City of Mandurah as part of the WA Food Security Project. The objectives of the first phase of the study were to assess the variety of foods available; the main types of foods purchased; the availability of healthy food choices; and factors influencing the choice of foods available in local food outlets.

## Methods

A food audit survey was developed (based on published literature and expert review) for distribution to all food outlet owners/operators in one lower socio-economic area within the City of Mandurah (n = 132 outlets identified from council records). A Letter of Introduction outlining the purpose of the food audit was mailed to the outlet owners followed by a person visit from their local Environmental Health Officer (EHO) who provided a paper-based survey to each owner/operator (as indicated in the letter). The EHO's were trained in the administration of the survey to ensure standardisation of instruction. A reply paid envelope addressed to the Principal Investigator to ensure confidentiality of responses. Ninety nine outlets completed the survey (response rate of 75%). (There was no significant differences between the types of outlets who did and did not respond.) The primary owner or operator of each food outlet was asked to provide information about: the variety of foods available in their outlet; the main types of foods purchased for preparation and resale; their knowledge and availability of healthy food choices; and factors influencing the choice of foods available to their clientele. (A copy of the food audit survey can be obtained from the principal author on request).

**Figure 1:**
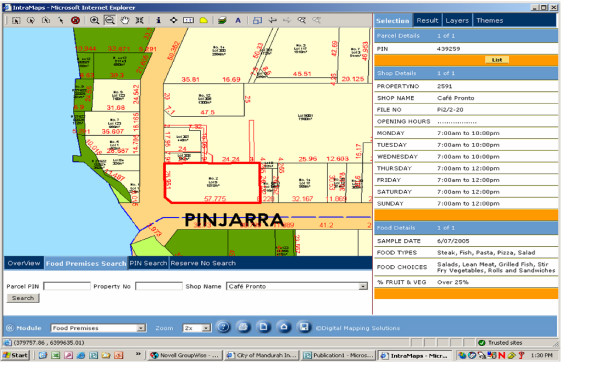
Example of food audit data mapped using the Geographical Information System.

All data from the surveys were analysed using univariate analysis methods. Ethics approval was granted from Curtin University of Technology and all results were de-identified prior to reporting. Data were also mapped via a Geographical Information System (GIS) to assess the location of each type of food outlet and the extent of the local population serviced. Figure 1 shows an example of some of the data associated with one outlet. The City of Mandurah has linked these data to their existing databases to inform the next phase of this project which will include a survey of local residents serviced by the outlets to assess their needs relating to food security. Results from the second phase will be available by the end of 2008.

### Ethical considerations

Ethics approval was received from the Curtin University Human Ethics Committee to conduct this research. This complies with the Helsinki Declaration for research conducted with humans. All participants provided written consent prior to being involved in this study and all results were de-identified prior to reporting.

The types of food outlets involved in the audit were local delicatessens (small food outlets that offer both perishable and non-perishable foods) and small takeaway outlets. No large supermarket chains or major fast food outlets were included.

## Results

Ninety nine outlets completed the food audit, therefore results are reported as percentages of outlets. Almost all outlets (90 to 97%) opened at least five days each week. The main clientele of the outlets were families (78%), tourists (59%), seniors (52%) or businessmen/women (44%).

A considerable amount of food available for purchase was pre-prepared (prepared before required and kept either refrigerated or in a bain maree), with more than half of the outlets pre-preparing the majority of their food. The main foods pre-prepared by outlets included sandwiches (n = 26%), salads (n = 21%), cakes (n = 15%) and hot foods (n = 14%). Sandwiches and rolls were the most popular items sold in the outlets surveyed (n = 51%) followed by pastries such as pies, sausage rolls and pasties (n = 33%).

The respondents were asked to detail the five healthiest foods they sold. Responses included sandwiches or rolls (n = 51%), salads (n- = 50%), fruit and vegetables (n = 40%), seafood (n = 27%), meats such as chicken (n = 26%) and hot foods such as curries, soups or quiches (n = 23%) (see Table 1).

**Table 1:** Healthy food choices reported by food outlets owners/operators (n = 99)

	**Health food choices**					**Number of outlets**
	
**Healthy Food Items**	**1^st^**	**2^nd^**	**3^rd^**	**4^th^**	**5^th^**	
Sandwich/roll	20	14	8	6	3	51
Salads	24	9	8	7	2	50
Fruit & veg	9	14	8	6	3	40
Seafood	8	6	9	3	1	27
Meats (chicken)	2	11	8	3	2	26
Hot food (curry, soup, quiche)	5	7	7	4	-	23
Vegetarian	5	5	4	3	1	18
Pasta (lasagne, potato bake)	1	3	5	4	-	13
Cakes/slices/sweets	4	5	1	1	-	11
Pastry (pie, s/roll, pasty, pizza)	4	2	-	2	-	8
Burgers	3	-	3	-	-	6
Other	5	2	2	4	1	14

Factors influencing the choice of foods offered at outlets included good service (n = 32%), consumer demand (n = 31%), cost effectiveness (n = 30%) and nutritional content (n = 2%). Several outlets offered food and drink combinations with most containing a sandwich, roll or burger plus a soft drink (n = 10–12%). Half of the outlets offered only white (n = 64%) or wholemeal bread or rolls (n = 50%). Less than one third of outlets offered multigrain (n = 28%), foccacia (n = 21%) or pita breads or rolls (n = 19%).

The most popular food items sold overall included sandwiches or rolls with fillings such as: chicken with or without cheese and salad (n = 80%), ham with or without cheese and salad (n = 63%) and tuna with or without salad (n = 26%) (see Table 2). Most outlets offered vegetable-based salads (n = 76%) and some offered a combination of salad and meats (n = 28%). Various dressings were available with the most common being mayonnaise (n = 26%), italian (n = 16%) or french (n = 14%).

**Table 2:** Most popular food items sold at food outlets (n = 99 outlets)

	**Five most popular foods**					**Number of outlets – most popular choice**
	
**Popular Food Items**	**1^st^**	**2^nd^**	**3^rd^**	**4^th^**	**5^th^**	
Sandwich/roll	21	11	13	4	2	51
Pastry (pie, s/roll, pasty, pizza)	8	11	6	5	3	33
Meats (chicken)	11	12	4	3	-	30
Seafood	16	5	4	2	2	29
Burgers	6	10	7	5	-	28
Cakes/slices/sweets	8	9	3	3	3	26
Hot food (curry, soup, quiche)	1	2	9	7	2	21
Salads	1	4	8	3	2	18
Pasta (lasagne, potato bake)	3	4	4	-	-	9
Fruit & veg	-	-	1	4	1	6

Outlets also reported on the hot foods available in bain-maries. Hot, savoury pastries such as pies, pasties, sausage rolls and dim-sims were the more popular products sold (n = 77%) followed by hot chips (n = 19%) (see Table 2).

Respondents were also asked about factors that affected their decision to stock fresh fruit and vegetables. Consumer demands (n = 58%), quality (n = 30%) and availability (n = 27%) were the main reasons given by outlets as to whether fresh fruit and vegetables were made available to their clientele (see Table 3). Carbonated drinks were the most popular beverage sold at most outlets (n = 88%) followed by bottled water (n = 62%) and flavoured milk drinks (n = 46%) (see Table 4).

**Table 3:** Factors influencing food outlets decisions to stock fresh fruit and vegetables

**Reason**	**Number of outlets (n = 99)**
Consumer demand	58
Consistency of Quality	30
Seasonal availability	27
Shelf life/spoilage	20
Storage (facilities, space, refrigeration)	20
Maintenance	7
Transportation issues	7

**Table 4:** Most popular beverages purchased weekly from food outlets

**Types of beverage**	**Three choices**			**Number of outlets**
	
	**1^st^**	**2^nd^**	**3^rd^**	
Carbonated drinks	78	10	-	88
Bottled water	-	13	49	62
Flavoured milk drinks	7	34	5	46
Fresh juices	1	24	9	34
Fresh milk	3	5	3	11

## Discussion and conclusion

This project involved 99 of a possible 132 food outlets from one lower socio-economical area in the City of Mandurah (response rate 75%). The majority of food outlets surveyed pre-prepared the majority of food sold and considered bread-based products to be healthy options regardless of the content of the filling or dressings used. Less than one third of the food outlets surveyed offered a choice of bread type other than white or wholemeal. High fat pastries and dressings were popular options offered, as were carbonated drinks and flavoured milks. These findings clearly indicate the need to investigate with local residents, the barriers and enablers associated with access to quality, healthy foods at a reasonable cost, within the study area.

For sustainable, positive change to food security in this locale within the City of Mandurah to be achieved, the level of training available to outlet owners/operators to provide healthy food options whilst maintaining a level of business profitability must be addressed. Furthermore, a significant number of outlet owners/operators must be prepared to support any initiatives implemented. It may be necessary for the City of Mandurah to provide incentives such as: reduced training costs; reduction of business fees to those involved for a period of time; or free advertising of outlets that achieve predetermined goals.

Future research should include an extension of the food audit to other areas within the local area to gain a better understanding of the extent of food security in the City of Mandurah. Profiling of the community perceptions of the cost, quality, and range of foods available in their local area would assist in determining the association between the socio determinants of health, dietary diversity and food security. Results from the extended food audit and community profiles could be used as the basis for the development of a multi-faceted intervention that will seek to improve the health of residents within the City of Mandurah.

## Competing interests

The author(s) declare that they have no completing interests.

## Authors' contributions

AM conducted research, analysed data, prepared report, GB and BM provided support to the development of the research proposal. All authors read and approved the final manuscript.

## Pre-publication history

The pre-publication history for this paper can be accessed here:


